# An audit of the fracture liaison service at Helen Joseph Tertiary Hospital, Johannesburg, South Africa

**DOI:** 10.1007/s11657-026-01774-x

**Published:** 2026-09-28

**Authors:** C. Van der Meer, R. Daya, Z. Bayat, L. S. Greenstein

**Affiliations:** 1 Department of Internal Medicine, Faculty of Health Sciences, School of Clinical Medicine, University of the Witwatersrand, Johannesburg, South Africa; 2 Division of Endocrinology and Metabolism, Department of Internal Medicine, Helen Joseph Tertiary Hospital, Johannesburg, South Africa; 3 Division of Geriatric Medicine, Department of Internal Medicine, Helen Joseph Tertiary Hospital, Johannesburg, South Africa

**Keywords:** Fragility fractures, Osteoporosis, Fracture Liaison Service, Secondary fracture prevention, South Africa, Resource-limited settings

## Abstract

***Summary*:**

Fragility fractures are increasing worldwide, yet osteoporosis often remains underdiagnosed and untreated. This study found a high burden of osteoporosis risk factors among patients enrolled in a South African fracture liaison service, highlighting the need for structured secondary prevention. These findings support expansion of fracture liaison services to improve long-term patient outcomes in resource-limited settings.

**Background:**

Fragility fractures (FF) driven by osteoporosis result in high morbidity and mortality and are a global health concern. Fracture liaison services (FLSs) are the gold standard for secondary prevention, yet data on their implementation and patient profiles in South Africa remain sparse. The Helen Joseph Tertiary Hospital (HJH) FLS was established to improve this care gap.

**Objectives:**

To describe the clinical and demographic characteristics, risk factors for osteoporosis and falls, osteoporosis risk scores, and management of patients enrolled in the FLS at HJH.

**Methods:**

This retrospective audit included 247 patients admitted to the orthopaedic ward and enrolled in the FLS between 03 October 2022 and 04 June 2024. Data on demographics, osteoporosis risk factors, investigations and management were analysed. Statistical significance was set at *p* < 0.05.

**Results:**

The majority of patients were female (74.5%), aged 60–89 years (81.4%). Hip fractures predominated (59.9%), specifically neck of femur (38.8%) and intertrochanteric fractures (21.1%). Although 82.5% of patients received calcium and vitamin D supplementation, only 27.8% received in-hospital bisphosphonate therapy (Zoledronic acid). Medication shortages accounted for 60% of cases in which Zoledronic acid was not administered, while 12.4% were ineligible due to advanced chronic kidney disease. In-hospital mortality was 5%, and median length of stay was 18 days (IQR 11–28). Vitamin D insufficiency was prevalent (41.1%) and did not differ by ethnicity. Significant gender differences were observed: men had substantially higher rates of smoking (73% vs 46%) and alcohol use (48% vs 23%). Inadequate physical activity was a risk factor in both genders. Fall risks included a history of previous falls (42.0%) and cognitive impairment (Mini-Cog <3; 39.6%). Patients with hip fractures had significantly higher FRAX scores than those with fractures at other sites (*p* < 0.05).

**Conclusions:**

This is the first South African Fracture Liaison Service study to report on fall risk, osteoporosis risk, and post-fracture management. Fragility fractures at Helen Joseph Hospital represent a substantial burden, with a high prevalence of clinical risk factors and notable gender-specific contributors, particularly alcohol use in men. While the FLS helps close the treatment gap, its impact is limited by inconsistent access to osteoporosis therapies. Improving outcomes in resource-constrained settings requires reliable medication supply and targeted interventions for fall prevention and modifiable risk factors.

## Introduction

Fragility fractures (FF) are an increasing health concern because they are associated with substantial morbidity, loss of independence and mortality, with the highest risk of adverse outcomes occurring within the 12–24 months after the initial fracture [1]. Osteoporosis is the leading underlying cause of FF, yet remains underdiagnosed and undertreated despite the availability of effective treatment [2, 3]. Fracture liaison services (FLS) are the international gold standard for secondary prevention of FF [4]. A FLS is a model of care designed to identify, assess and manage patients with FF through a coordinated multidisciplinary approach that spans both the acute setting and long-term follow up. This aims to reduce the risk of subsequent fractures [5–7]. International studies demonstrated that FLS improves bone mineral density (BMD) testing, the initiation of osteoporosis medication, and long term adherence, while decreasing further fractures and mortality [8]. Research shows an increase in initiation of bisphosphonates of 72.1% where FLS is available compared to 45.1% when the service was not available [9].

Implementation and outcomes vary across settings due to differences in healthcare infrastructure, access to diagnostic modalities for osteoporosis, and patient factors affecting follow up. In South Africa, the burden of osteoporosis and FF is increasing, but remains under researched compared to high income countries [10]. Data on osteoporosis prevalence, risk factors and uptake of treatment is limited and FLS models are still in early stages of development in most centres [11]. Helen Joseph Tertiary Hospital (HJH) in Gauteng, South Africa, established an FLS to provide structured acute and long-term management for patients with FF. Understanding the demographics and risk factor profile of patients with FF managed through the FLS is crucial to optimise service delivery and align the programme with international standards.

This study aimed to describe the demographic, clinical, laboratory, and risk factor characteristics of patients with FF managed through the FLS at HJH, and to explore associations between risk factors, fracture characteristics, and FRAX (Fracture Risk Assessment Tool) scores.

## Methods

### Study design

This retrospective audit included the first 247 patients enrolled in the FLS at HJH, the first service of its kind in the Gauteng public sector. The audit covered a 20-month period from 03 October 2022 to 04 June 2024, representing the initial phase following service implementation. Data were extracted from a standardised FLS assessment form routinely completed for all patients, capturing demographic, clinical, laboratory, and risk factor information, inpatient management and outcomes.

### Setting

Helen Joseph Tertiary Hospital is an academic public sector hospital in Gauteng, South Africa, serving a population of approximately one million people. The FLS was established to identify, assess, and manage patients presenting with FF, with the aim of preventing refracture and improving osteoporosis management. The FLS is based on five pillars including patient identification, screening for secondary causes of osteoporosis, initiating treatment, providing follow up care, and research. Patients admitted with FF were screened daily by the FLS team for risk factor assessment, treatment initiation and coordination of follow-up care. Inpatient care was provided by the departments of geriatrics and orthopaedics, while outpatient follow up care was provided by the divisions of Endocrinology and Geriatrics.

### Participants

Eligible patients were all in-patients admitted with fragility fractures and enrolled in FLS. Fragility fractures were defined as fractures resulting from low-energy trauma, such as a fall from standing height or less. In accordance with National Osteoporosis Foundation of South Africa(NOFSA) Clinical guidelines [12], eligible patients included postmenopausal females irrespective of age, and males aged 50 or older. Patients with pathological fractures, fractures resulting from high-energy trauma, premenopausal females, men younger than 50 years and patients managed exclusively as outpatients were excluded as they are not automatically enrolled into the FLS. A total of 247 patients were included in the audit.

### Data collection

Data were extracted retrospectively from standardised FLS patient assessment forms and corresponding medical hospital records.

Demographic characteristics included age, gender and ethnicity.

Clinical characteristics included fracture details: site, type and laterality. Falls history and fall risk factors included previous falls (>2 in past 12 months), gait instability, prior stroke, reduced clinical muscle strength, previous mobility (independent, use of a walking aid or immobile), dementia, visual or hearing impairment, abnormal cardiac examination (including atrial fibrillation, aortic stenosis, aortic sclerosis), bladder or bowel incontinence, proprioceptive or foot problems. Cognitive assessment by calculating a Mini-Cog score. Co-morbidities and any documentation of chronic conditions. Lifestyle factors including smoking, alcohol use (>3 units/day), physical activity (<150 min/week), low body weight (BMI < 20 kg/m^2^) caffeine intake (>3 cups/day), dietary calcium intake. Genetic/familial risk factors: family history, ethnicity and prior fragility fractures. Medication history which included current and prior medications, including osteoporosis therapy.

Laboratory investigations were performed on initial contact with the FLS, prior to surgery and included kidney function (estimated Glomerular Filtration Rate) [eGFR], vitamin D levels, calcium levels, full blood count (FBC) and thyroid-stimulating hormone (TSH). Although considered to be clinically relevant, erythrocyte sedimentation rate (ESR), parathyroid hormone (PTH), serum protein electrophoresis (SPEP) were excluded from the analysis due to significant missing data (49%, 36% and 60% respectively).

Osteoporosis Assessment included Dual-energy X-ray absorptiometry (DEXA) scans performed during the index hospital admission or within one year of the fragility fracture, where available. DEXA results were available in 21% of patients. Fracture risk was assessed using the FRAX tool, including both the 10-year probability of a major osteoporotic fracture and hip fracture. To ensure consistency across the study cohort, FRAX scores were calculated without bone mineral density (BMD) because of limited availability of DEXA results. All DEXA scans were performed using a Hologic QDR densitometer.

Treatment outcomes were measured by the initiation of Zoledronic acid, calcium and/or vitamin D supplementation, time to fracture liaison service (FLS) enrolment and length of hospital stay.

Mortality was measured by in-hospital deaths during admission.

Missing data were handled using available-case analysis. Participants with missing values were excluded only from the relevant calculation, so denominators varied according to the available data for each variable. Where applicable, the denominator used was reported.

### Outcome measures

The primary outcome was descriptive, comprising the demographic and clinical profile of patients enrolled in the FLS. Secondary outcomes included findings from osteoporosis investigations (including evaluation for secondary causes), rates of osteoporosis treatment initiation, and associations between risk factors, fracture characteristics, and FRAX scores.

### Data analysis

Data were analysed using descriptive and inferential statistics. Categorical variables were summarised as frequencies and percentages, while continuous variables were reported as means with standard deviations (SD) or medians with interquartile ranges (IQR), depending on distribution. Selected study variables were compared between genders using the chi-squared test, with Fisher’s exact test applied for 2 × 2 tables or where chi-squared assumptions were not met, comparisons of categorised (Vitamin D levels and self-reported ethnicity) were done similarly. Associations between the presence or absence of any non-modifiable (genetic/ethnic or comorbidity) risk factors and fracture site were analysed as described above, while associations with FRAX data were tested using the Wilcoxon rank sum test. The relationship between FRAX hip results and fracture site (NOF vs. Intertrochanteric vs. other) was assessed with the Kruskal-Wallis test. All analyses were performed using SAS version 9.4 for Windows, with statistical significance set at *p* < 0.05.

Data were stored on a double encrypted system with dual password protection that was only available to the investigators and data analyst.

## Results

### Demographic characteristics

A total of 247 patients were seen by the FLS between 03 October 2022 and 04 June 2024. Table 1 shows the breakdown of the demographics within this cohort. The mean age was 71.8 years (SD 11.9), with the majority of patients aged 60 years and older. Females comprised 74.5% of the cohort. Ethnicity was self-identified, with 45% Caucasian patients, 22.7% African, 22.3% Coloured, and 8.7% Indian/Asian.

**Table 1:** Table of demographics

Characteristic	Category	Female	Male	*p*-value
		*n* (%)	*n* (%)	
Age	40–49 year	6(3.3)	0(0.0)	0.10
	50–59 year	21(11.4)	7(11.1)	
	60–69 year	52(28.3)	17(27.0)	
	70–79 year	56(30.4)	28(44.4)	
	80–89 year	37(20.1)	11(17.5)	
	90–99 year	12(6.5)	0(0.0)	
Site of fracture (*n* = 242)	Neck of femur	66(35.9)	28(44.4)	0.46
	Intertrochanteric	37(20.1)	14(22.2)	
	Humerus	16(8.7)	6(9.5)	
	Ankle	15(8.2)	2(3.2)	
	Femur	15(8.2)	2(3.2)	
	Forearm	14(7.6)	2(3.2)	
	Other	20(10.9)	8(12.7)	
**Risk factors**				
**Lifestyle**	Inadequate physical activity (<150 min/week)	97(53.3)	32(52.5)	>0.99
	Smoking	84(46.2)	45(72.6)	**0.0004**
	Diet poor in calcium	75(41.4)	32(52.5)	0.14
	Alcohol use (>3/day)	41(22.5)	30(48.4)	**0.0002**
	Low BMI (<20 kg/m^2^)	47(27.5)	16(27.1)	>0.99
	Excess caffeine use (>3/day)	30(16.6)	14(23.3)	0.25
**Risk factor for falls**	Previous fall	73(40.3)	29(46.8)	0.38
	>2 falls per year	45(24.9)	24(38.7)	**0.050**

Bold values indicate significance as set out by *p* < 0.05

### Fracture characteristics

**Fig. 1:**
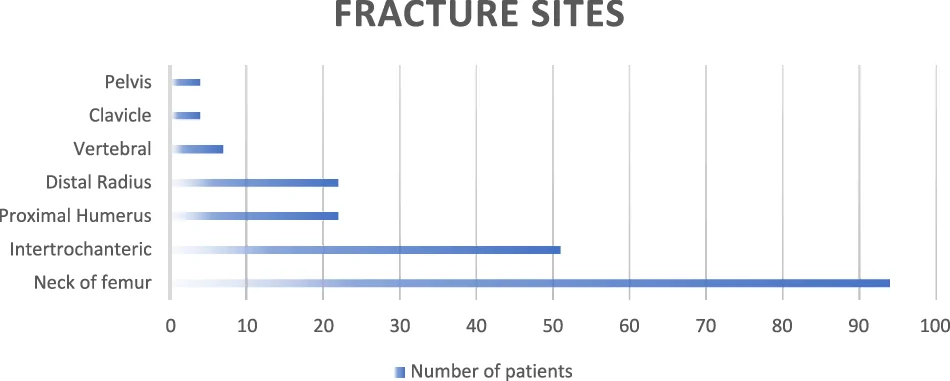
Distribution of fracture sites among patients with fragility fractures

Hip fractures were the most common fracture type, with intertrochanteric fractures (21.1%) and neck of femur fractures (38.8%) predominating. Other fracture sites included the distal radius (7.0%), proximal humerus (9.1%), pelvis (4.5%), and vertebrae (2.9%). Laterality was evenly distributed, with 50.8% of hip fractures occurring on the left side. The distribution of fracture types is illustrated in Fig. 1.

### Clinical features and co-morbidities

#### Co-morbidities

**Fig. 2:**
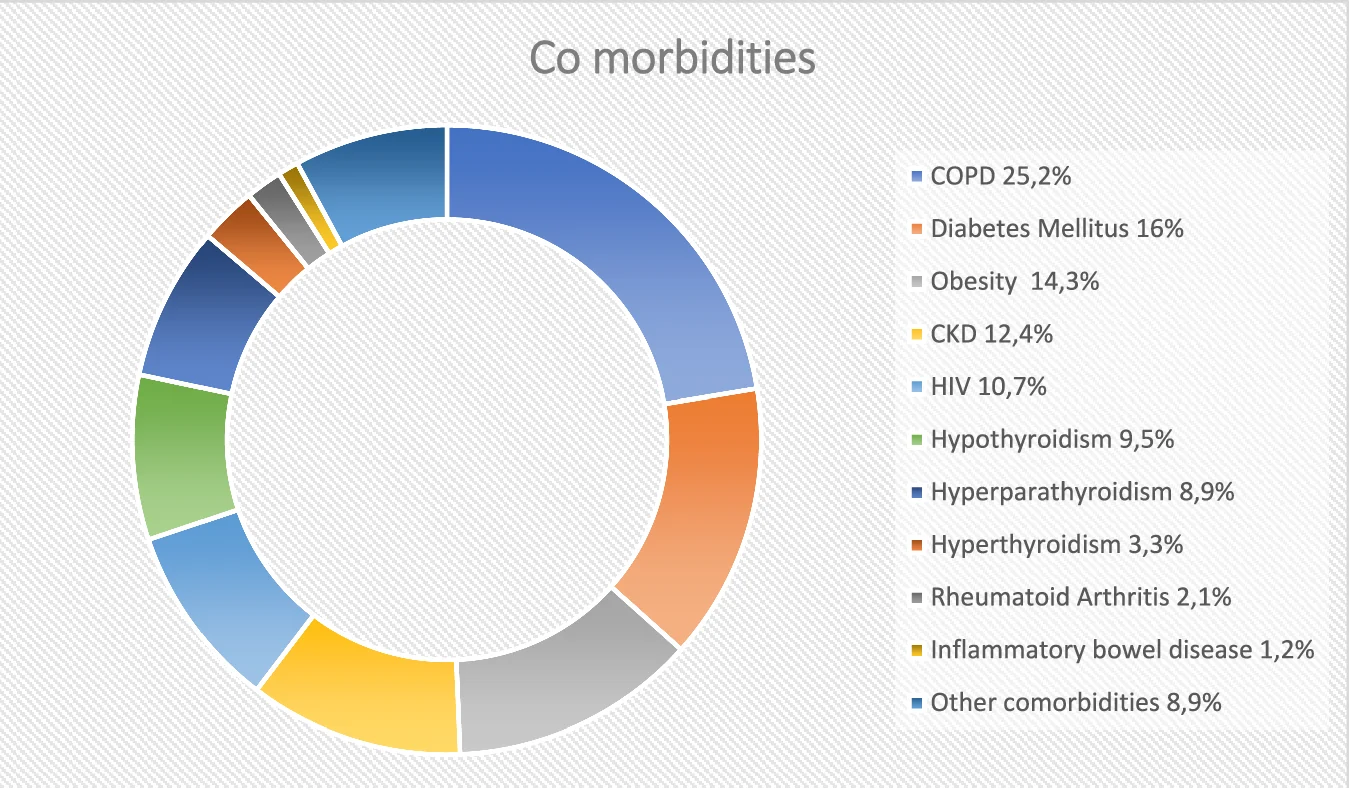
Distribution of comorbidities amongst patients with fragility fractures

Co-morbidities were common, (Fig. 2) with chronic obstructive pulmonary disease (COPD) (25.5%) and diabetes mellitus (16.0%) being the most prevalent. Obesity was documented in 14.3% of patients.

#### Fracture history

A history of previous fragility fracture was present in 27.2% of patients, highlighting a substantial burden of recurrent fractures within this cohort.

#### Falls and cognitive risk

**Fig. 3:**
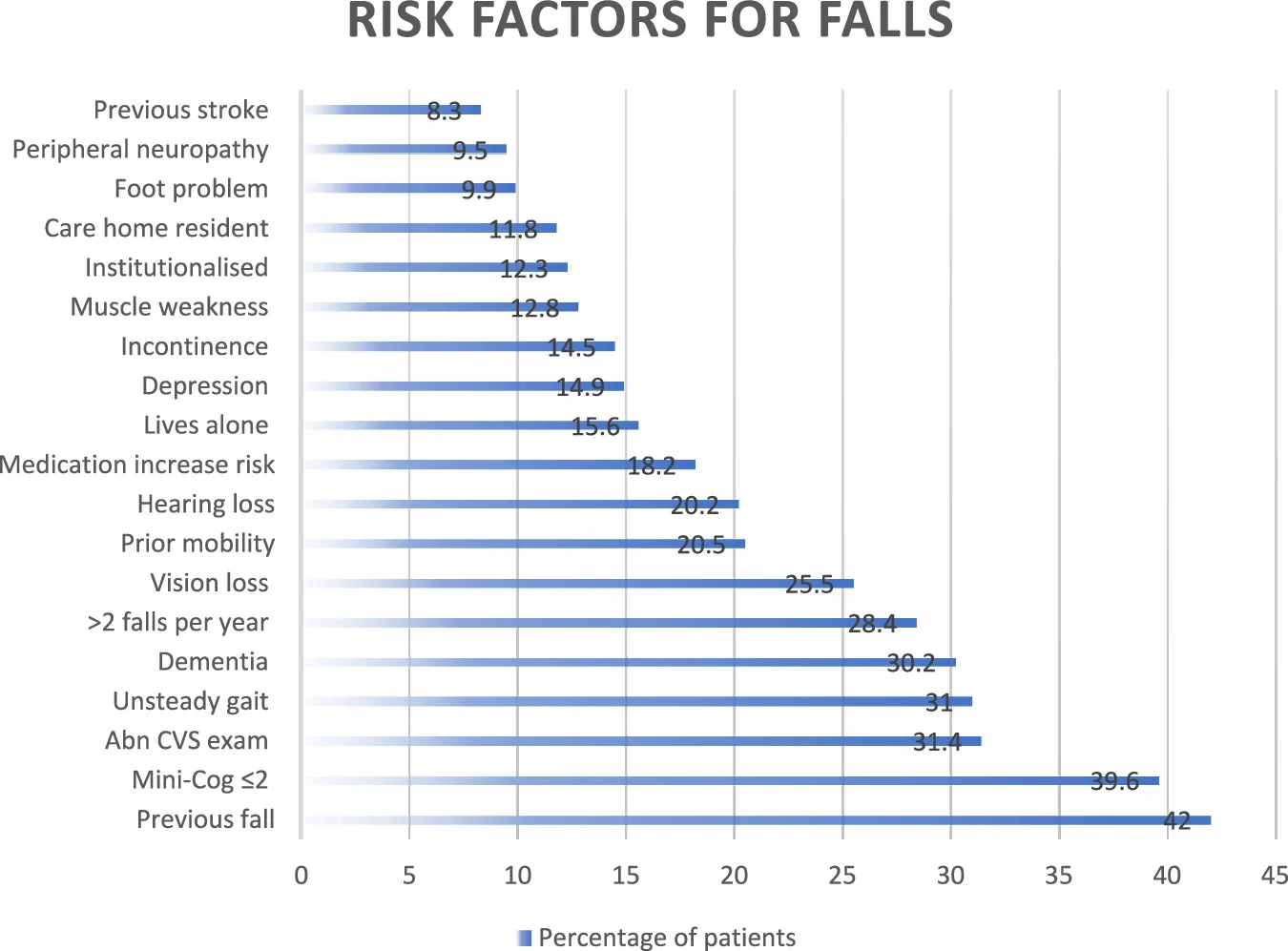
Risk factors for falls

Falls risk was high, with  42% of patients reporting at least one fall in the preceding year, with no significant difference between men and women. The distribution of falls risk factors is shown in Fig. 3. Mini-cog screening identified possible cognitive impairment in 39.6% of those assessed.

#### Lifestyle risk factors

Lifestyle risk factors were frequent, including smoking (52.9%) and physical inactivity (53.1%). Males had substantially higher rates of smoking (73% vs 46%) as compared to their female counterparts. Alcohol consumption of more than 3 units per day, was found in 21.9% of all patients, but was substantially higher among men (48.0%).

#### Medication exposure and treatment gap

Exposure to medications associated with increased bone resorption (including long-term corticosteroids, warfarin, antiepileptics, chemotherapy, and excess thyroid hormone) was identified in 19% of patients. Despite this, only 3.3% of patients were receiving osteoporosis treatment prior to the index fracture.

### Laboratory findings

**Fig. 4:**
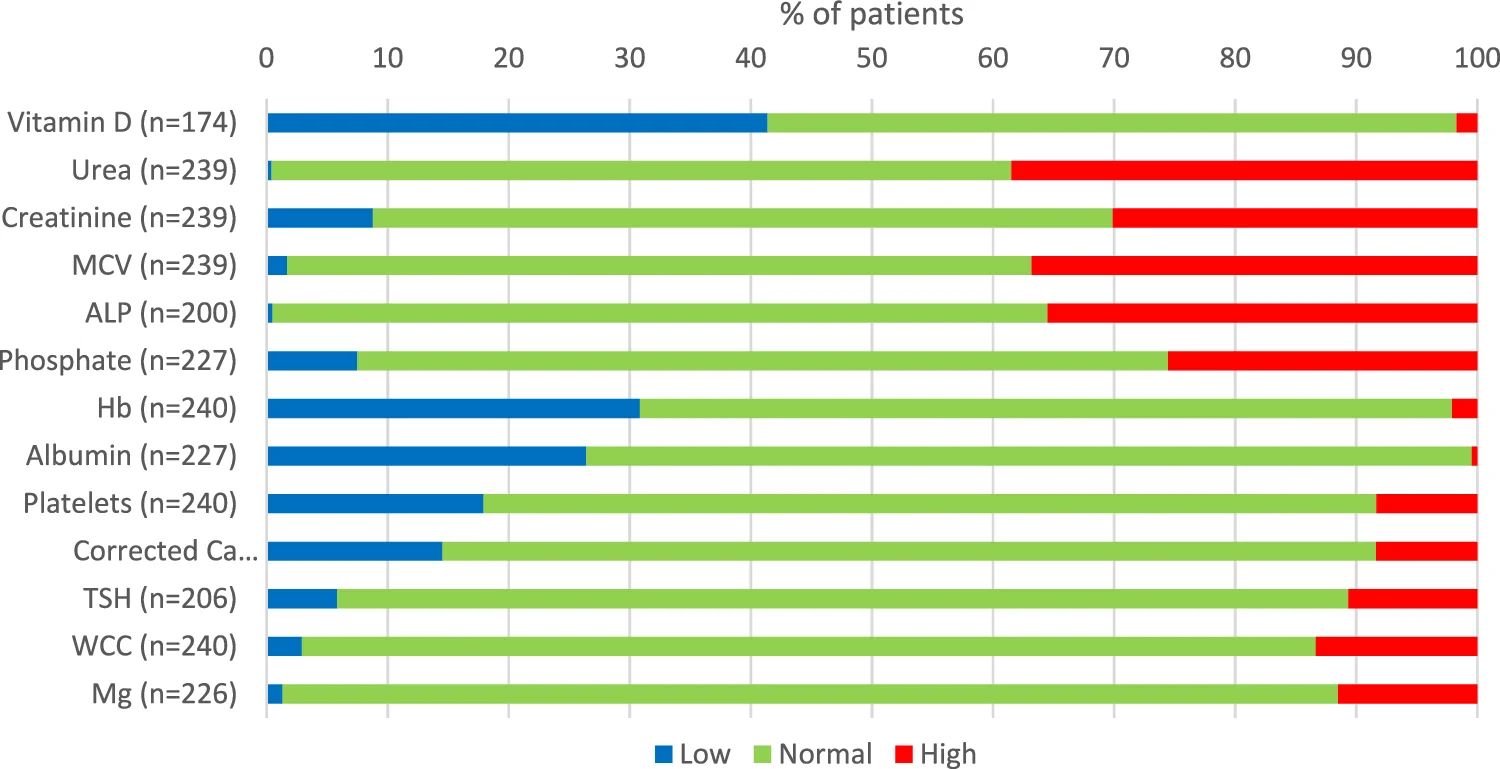
Laboratory findings amongst patients in this cohort

Figure 4 shows the laboratory findings of this study population. Renal impairment was frequent, with 11.3% of patients having chronic kidney disease (CKD) as measured by estimated Glomerular Filtration Rate (eGFR) and 35.6% presenting with acute kidney injury (AKI). Vitamin D insufficiency was documented in 41.1% of patients with no differences between ethnic groups (*p* = 0.64).

### Osteoporosis assessment

According to the World Health Organization (WHO), osteopenia is defined by a T-score between –1.0 and –2.5, while a T-score less than –2.5 indicates osteoporosis [13]. Among those assessed with DEXA, bone mineral density (BMD) was in the osteoporotic range in 42.2% and in the osteopenic range in 35.5%. FRAX scores (calculated without BMD), indicated a high fracture risk, with a median 10-year probability of major osteoporotic fracture of 9.8% (IQR 4.4–19.0) and hip fracture of 4.1% (IQR 1.5–11.0). Patients with at least one non-modifiable risk factor had significantly higher FRAX major osteoporotic fracture scores (median 10.0; IQR 4.8–20.0) compared with those without non-modifiable risk factors (median 5.1; IQR 2.5–12.0).

### Management and treatment outcomes

The median time from admission to assessment by the FLS was two days (IQR 1–4). Calcium and/or vitamin D supplementation was prescribed to 82.5% of all patients, who either had a documented deficiency or reported a dietary deficiency, and 27.8% received Zoledronic acid in-hospital prior to discharge (Table 2). Among those who did not receive Zoledronic acid, 12.4% were ineligible due to advanced CKD (eGFR <35 mL/min/1.73 m²), while the majority (60%) did not receive treatment because of medication supply shortages. Those with severe renal impairment were unable to receive alternative therapies, such as RANK ligand inhibitors or teriparatide, due to the unavailability in the state sector.

**Table 2:** Percentage of treatment received

	*n* (Percentage %)
Pre fracture medication (*n* = 243)	8 (3.3)
Previously on OP medication (*n* = 244)	10 (4.1)
Zoledronic acid received (*n* = 198)	55 (27.8)
Calcium/Vitamin D given (*n* = 183)	151 (82.5)
In-hospital death (*n* = 239)	12 (5.0)

The median length of hospital stay was 18 days (IQR 11–28). In-hospital mortality was 5%, largely related to pulmonary embolism and sepsis-related complications.

### Associations between risk factors and outcomes

Significant associations were observed between age and fracture site, with hip fractures more common in patients aged ≥ 70 years (*p* < 0.05). Female gender was strongly associated with higher FRAX scores for both major osteoporotic and hip fracture risk (*p* < 0.01). Vitamin D insufficiency was significantly associated with reduced BMD and higher FRAX risk scores (*p* < 0.05). Smoking and alcohol use were also associated with increased fracture risk, though these associations did not reach statistical significance.

## Discussion

### Principal findings

This study provides one of the first detailed descriptions of patients managed through a Fracture Liaison Service (FLS) in a state hospital in Gauteng, South Africa, and is the first to incorporate detailed assessment of falls risk and a wide range of clinical risk factors. It highlights the substantial burden of untreated osteoporosis, high falls risk, and multimorbidity among individuals presenting with fragility fractures.

The predominance of older women is consistent with the well-recognised epidemiology of FF in postmenopausal women [1]. Hip fractures were the most frequent presentation, reflecting the combined impact of osteoporosis and falls in this population. The high prevalence of co-morbidities, prior FF, and vitamin D insufficiency underscores the medical complexity of patients presenting with fractures in this setting. Despite evidence of significant skeletal fragility, very few patients were receiving osteoporosis therapy prior to fracture, highlighting a substantial treatment gap. While the FLS enabled identification of high-risk patients and initiation of therapy, treatment implementation was constrained by medication shortages and limited availability of alternative therapies.

### Comparison with existing literature

The demographic profile of this cohort mirrors international findings, where FF predominantly affects older women [2, 3]. African patients comprised a smaller proportion of the study population compared with Caucasian patients (22.7% vs. 45%). This pattern is consistent with previous studies suggesting that there is a lower prevalence of osteoporosis among individuals of African ancestry [14]. Other risk factors identified throughout literature that were also observed in our study include older age, lower BMD, a previous history of FF and recurrent falls [3, 8]. The mean age of 71.8 years is consistent with South African and global studies, where fracture incidence increases sharply beyond the seventh decade of life [15, 16]. The predominance of hip fractures aligns with data from the Global Burden of Disease study, which highlights hip fractures as the most serious and costly consequence of osteoporosis [17].

The high prevalence of prior falls, cognitive impairment and multimorbidity observed in these patients, highlights the multifactorial vulnerability of patients presenting with FF. In a Norwegian study, falls preceded fractures in 83% of patients, whilst 38% of community dwelling patients had pre-existing cognitive impairment [18]. Smith et al. showed 1.53-fold risk of fall resulting in injuries amongst individuals with cognitive decline in middle- to low- income countries [19]. Leslie et al. demonstrated a positive dose-response relationship between fall frequency and the risk of major osteoporotic fracture [20]. This supports our finding that patients with two or more falls per year trend towards increased risk of FF (*p* = 0.050). These findings reinforce the importance of integrated fracture prevention strategies that address bone health and fall risks.

The relatively high prevalence of comorbid conditions in this study is consistent with previous studies showing that multimorbidity is an important determinant of outcome following a fragility fracture [21]. Cognitive impairment, observed in 39.6% of patients, is known to increase falls risk and complicate rehabilitation [22, 23]. Similarly, vitamin D insufficiency, present in 41.1% of this cohort, has been widely reported in both high- and low-income settings, reflecting limited sun exposure, nutritional deficiencies, and underlying comorbid disease [10]. In this study, vitamin D insufficiency did not differ significantly across ethnic groups, indicating no observable ethnic variation within this cohort and highlighting the need to explore other contributing factors in this setting.

A notable finding in this cohort was the markedly higher prevalence of smoking and alcohol use among men compared with women. While Søgaard et al. reported similar patterns in men aged 39–59, they found no such associations in men older than 60 years [24]. This supports previous observations that men presenting with fragility fractures often have a greater burden of modifiable lifestyle risk factors and may represent a particularly vulnerable subgroup requiring targeted prevention strategies [10, 25, 26].

Although osteoporosis is traditionally considered less prevalent in African populations, emerging data suggest that FF in African patients may be under-recognised due to lower rates of diagnostic testing and referral [27, 28]. The ethnic distribution observed in this study may therefore reflect patterns of healthcare access and referral rather than true disease prevalence.

The FRAX results in this population are consistent with other studies showing elevated 10-year fracture risks among elderly hospitalised patients [29]. The association of female sex, vitamin D insufficiency, and advanced age with higher risk scores reinforces established risk factors for osteoporosis [14, 16].

The median length of hospital stay was 18 days (IQR 11–28), more than double reported in high income countries such as the United Kingdom and the United States of America, where average hospital stays following FF range from 5 to 8 days. Prolonged hospitalisation may contribute to increased healthcare costs and reflects differences in health system resources [30, 31].

Low rates of in-hospital treatment initiation, together with evidence of poor long-term adherence in resource-limited settings [32], suggest gaps at multiple stages of care, including both initiation and ongoing follow-up. Further research should focus on improving follow-up programmes and understanding barriers to sustained treatment.

Pre-FLS implementation data were not available at our institution, precluding a direct comparison of outcomes before and after implementation of the service. However, this study provides an important baseline against which future improvements in referral pathways for both inpatients and outpatients, service delivery, access to osteoporosis investigation and treatment, and overall FLS performance can be evaluated as the service continues to mature.

Zoledronic acid has been proven to reduce subsequent FF in 76% of patients [33, 34]. Severe drug shortages observed in our study thus impact the expected outcomes of FLS. Reid et al. showed that there is still benefit in single dose Zoledronic acid administration to reduce fractures by 32%, however, HORIZON Pivotal Fracture Trial demonstrated approximately 70% reduction in vertebral fractures and 41% reduction in hip fractures with annual Zoledronic acid [34, 35].

### Strengths and weaknesses

The strengths of this study include the systematic collection of data from a large cohort in a real-world clinical setting, providing valuable insights into fragility fracture risk factors and management in a South African tertiary hospital. These findings allow for the identification of risk factors and gaps in care which can be a target for further interventions and service improvements.

However, a few limitations should be acknowledged. First, the retrospective design relies on the accuracy and completeness of clinical records. Secondly, follow-up data on longer-term outcomes such as refracture rates, medication adherence, and functional recovery were not analysed. Thirdly, as a single-centre study, the findings may not be generalisable to other hospitals or regions, particularly rural areas with more limited healthcare access and resources. Lastly, as this study excludes patients exclusively managed as outpatients, we acknowledge it likely underestimates distal radius fractures commonly managed without admission. Limited availability of DEXA scanning in only 21% of patients during or one year following admission for ff, meant that BMD measurements were not available for a majority of patients and could not be analysed in this cohort. This limitation was due to the availability of DEXA being limited to 3 inpatients per day and 2 outpatients only.

### Contribution to knowledge and implications

This study demonstrates that a FLS implementation in a tertiary public sector hospital in South Africa is feasible and provides a coordinated model of care for patients with FF. The findings highlight the need for routine screening and early intervention, especially in older females with multiple co-morbidities. The high prevalence of vitamin D insufficiency suggests that supplementation should remain a cornerstone of management. Furthermore, the observed associations between risk factors and fracture outcomes provide evidence to guide targeted interventions.

For clinicians and policymakers, the results underscore the need to strengthen secondary fracture prevention programmes in South Africa. Investment in FLS models of care has been shown internationally to improve treatment initiation, reduce refracture rates, and lower healthcare costs. Allowing for improvement in availability of treatment and access to investigations such as DEXA may improve outcomes for FLS patients. Simple falls risk screening and risk factor screening should be standard in patients at risk of osteoporosis and should be coupled with a multidisciplinary team intervention when patients are identified as high risk.

### Unanswered questions and future research

Future research should examine long-term outcomes of patients managed through the FLS, including refracture rates, mortality, and functional recovery as well as direct comparison between groups who were and were not affected by drug shortages. Prospective studies are needed to assess treatment adherence, follow up and cost-effectiveness in the South African context. Expanding the FLS model to district hospitals and integrating it into primary care may also be valuable areas for study. Early intervention and reduction of modifiable risk factors, such as alcohol use, smoking, poor calcium intake can be addressed at every level of care and thus prevent further complications such as FFs.

## Conclusions

The HJH FLS successfully managed patients with osteoporotic FF in its first 20 months, demonstrating feasibility and utility of this model in a South African tertiary hospital. This study highlights a substantial burden of fragility fractures occurring in medically complex older adults with high fall risk, Vitamin D insufficiency, and very low levels of pre-fracture osteoporosis treatment. We found important gender-specific contributors including alcohol use in men. The FLS demonstrated effectiveness in identifying, assessing, and initiating treatment for patients with FF, with high rates of calcium and vitamin D supplementation in patients with deficiencies. However, initiation of bisphosphonate therapy remained suboptimal.

A multi-disciplinary FLS allows early identification and treatment of these high-risk patients, but treatment implementation is constrained by medication availability. These findings provide important local data to guide secondary fracture prevention strategies in South Africa. Future research should focus on long-term outcomes, treatment adherence, and cost-effectiveness to inform sustainable models of osteoporosis care in resource-limited settings. It also highlights the systemic issues, including shortages in investigations and treatment options, and therefore emphasise the use of other tools such as FRAX to aid in resource-limited settings.
